# Repeated oxytocin prevents central sensitization by regulating synaptic plasticity via oxytocin receptor in a chronic migraine mouse model

**DOI:** 10.1186/s10194-021-01299-3

**Published:** 2021-07-27

**Authors:** Yunfeng Wang, Qi Pan, Ruimin Tian, Qianwen Wen, Guangcheng Qin, Dunke Zhang, Lixue Chen, Yixin Zhang, Jiying Zhou

**Affiliations:** 1 Department of Neurology, The First Affiliated Hospital of Chongqing Medical University, 1st You Yi Road, Yuzhong District, 400016 Chongqing, China; 2 Department of Neurology, Nanchong Central Hospital, Nanchong, China; 3 Laboratory Research Center, The First Affiliated Hospital of Chongqing Medical University, Chongqing, China

**Keywords:** Chronic migraine, Oxytocin, Oxytocin receptor, Central sensitization, Synaptic plasticity

## Abstract

**Background:**

Central sensitization is one of the characters of chronic migraine (CM). Aberrant synaptic plasticity can induce central sensitization. Oxytocin (OT), which is a hypothalamic hormone, plays an important antinociceptive role. However, the antinociceptive effect of OT and the underlying mechanism in CM remains unclear. Therefore, we explored the effect of OT on central sensitization in CM and its implying mechanism, focusing on synaptic plasticity.

**Methods:**

A CM mouse model was established by repeated intraperitoneal injection of nitroglycerin (NTG). Von Frey filaments and radiant heat were used to measure the nociceptive threshold. Repeated intranasal OT and intraperitoneal L368,899, an oxytocin receptor (OTR) antagonist, were administered to investigate the effect of OT and the role of OTR. The expression of calcitonin gene-related peptide (CGRP) and c-fos were measured to assess central sensitization. N-methyl D-aspartate receptor subtype 2B (NR2B)-regulated synaptic-associated proteins and synaptic plasticity were explored by western blot (WB), transmission electron microscope (TEM), and Golgi-Cox staining.

**Results:**

Our results showed that the OTR expression in the trigeminal nucleus caudalis (TNC) of CM mouse was significantly increased, and OTR was colocalized with the postsynaptic density protein 95 (PSD-95) in neurons. Repeated intranasal OT alleviated the NTG-induced hyperalgesia and prevented central sensitization in CM mouse. Additionally, the OT treatment inhibited the overexpression of phosphorylated NR2B and synaptic-associated proteins including PSD-95, synaptophysin-1 (syt-1), and synaptosomal-associated protein 25 (snap25) in the TNC of CM mouse and restored the abnormal synaptic structure. The protective effect of OT was prevented by L368,899. Furthermore, the expression of adenylyl cyclase 1 (AC1)/ protein kinase A (PKA)/ phosphorylation of cyclic adenosine monophosphate response element-binding protein (pCREB) pathway was depressed by OT and restored by L368,899.

**Conclusions:**

Our findings demonstrate that repeated intranasal OT eliminates central sensitization by regulating synaptic plasticity via OTR in CM. The effect of OT has closely associated with the down-regulation of AC1/PKA/pCREB signaling pathway, which is activated in CM model. Repeated intranasal OT may be a potential candidate for CM prevention.

## Introduction

Chronic migraine (CM), with the criteria of ≥ 15 headache days per month and the migraine headache at least 8 days per month, has a high disability rate and large disease burden [1]. According to epidemiologic studies and clinical observations, approximately 3 % of episodic migraine (EM) patients progress to CM each year [2, 3]. However, available management approaches are scarce, due to complex pathogenesis and poor treatment response [4].

Oxytocin (OT), a nine amino acid peptide released from the paraventricular nucleus and supraoptic nucleus of the hypothalamus, induces uterine contractions, lactation, and regulates social behavior. Recent studies have identified OT as an endogenous modulator of pain and confirmed that paraventricular neurons can project to laminae I and outer II of the medullary trigeminovascular neurons [5–8], which is a core structure in headache chronification according to previous studies [9, 10]. In addition, OT exerts its effect via oxytocin receptors (OTRs), which is G-protein-coupled receptors [11]. Other researches find that OTRs are extensively expressed throughout the rat trigeminovascular system, including the trigeminal ganglia (TG) and trigeminal nucleus caudalis (TNC) [12, 13]. Another research demonstrates a slightly elevated OT level in blood samples in interictal CM patients [14]. These results imply that OT and OTR may regulate headache through their actions in the central and peripheral regions of the trigeminovascular system.

For the effect of OT, one study indicates that chronic administration of OT produces long-term observational fear in mice [15]. Moreover, a study on autism spectrum disorder shows that repeated OT administration could reduce the transcript expression of N-methyl D-aspartate receptor subtype 2B (NR2B) in the medial prefrontal cortical of C57BL/6 male mouse, compared with a single dose of OT administration [16]. A recently published review concludes that repeated OT administration closely resembles long-term treatment, and may cause changes in the structure and function of related brain regions, leading to different physiological effects [17]. Thus, the repeated administration of OT may be a potential therapeutic tool for CM.

Currently, central sensitization is considered to be one of the main mechanisms of CM [18, 19]. As EM progresses to CM, the experience of sustained or interictal cutaneous allodynia and prolonged sensitization in the TNC can be defined as central sensitization. One of the neuronal changes that underlies central sensitization is the recruitment and activation of N-methyl-D-aspartate (NMDA) receptors in the dorsal horn [19, 20]. In addition, phosphorylated NR2B may regulate the trafficking and synaptic localization of NMDA receptors to maintain their activation [21]. Other evidence has shown that the activation of Ca^2+^/calmodulin-stimulated adenylyl cyclase 1 (AC1) and protein kinase A (PKA) is essential for the activation of NMDA receptors [22]. PKA actives Src family kinases to phosphorylate the NR2B subunit [22]. In addition, PKA also drives the phosphorylation of CREB (pCREB), promotes the expression of genes including c-fos and synaptic NMDARs, and results in the long-lasting strengthening of synapses [9]. Therefore, the AC1/PKA/pCREB pathway can regulate the activity of NMDA receptors and then cause changes in synaptic plasticity. Our previous works have shown that the upregulation of the phosphorylated NR2B subunit is involved in synaptic plasticity and central sensitization in CM rats [23].

As for the effect of OT on migraine, one preclinical experiment demonstrated that intranasal OT could inhibit the responses of TNC to noxious stimulation [24]. In addition, their following clinical study implied that long time dosing of intranasal OT might reduce the frequency of chronic and high frequency migraineurs [24]. Therefore, the study about the effect and the related mechanism of repeated OT in CM is necessary. In our study, we hypothesized that the repeated OT treatment might affect the central sensitization and synaptic plasticity in CM mouse. Our research found that the expression of OTR in the TNC was significantly increased in the NTG-induced CM mouse model. Repeated OT alleviated central sensitization through restoring aberrant synaptic plasticity via OTR in CM. In addition, we also demonstrated that the down-regulation of AC1/PKA/pCREB signaling pathway was partially involved in the protective effect of OT. Thus, repeated intranasal OT administration may be a potential tool for CM prevention.

## Methods

### Animals

 All experimental procedures were approved by the Ethics Committee of the Department of Medical Research from the First Affiliated Hospital of Chongqing Medical University. Given the notable sex differences in migraine prevalence and the fluctuation in OT and estrogen during the menstrual cycles of females, male mice were selected to limit these discrepancies. A total of 120 male C57BL/6 mice weighing 20 ~ 30 g and 6 ~ 8 weeks of age were provided by the Experimental Animal Center of Chongqing Medical University (Chongqing, China). All animals were maintained in a room with a standard experimental environment and an alternating 12-hour light/dark cycle. To avoid social isolation stress, all mice were housed (6 per cage) in groups. Food and water were provided ad libitum. All animals were acclimatized to the environment for at least 1 week and then randomly assigned to different experimental groups. All animal procedures were conducted by the National Institutes of Health Guide for the Care and Use of Laboratory Animals.

### CM mouse model

The recurrent NTG-induced CM model was established according to the previous study [25]. A solution of 5.0 mg/ml NTG (Beijing Reagent, China) dissolved in 30 % alcohol, and 30 % propylene glycol was prepared. Before injection, NTG was freshly diluted in 0.9 % saline at 1 mg/ml.

After the nociceptive threshold test, mice received intraperitoneal (i.p.) injections of diluted NTG or an equal volume of vehicle (0.9 % saline ) every 2 days 5 times. Saline (0.9 %) was used for the vehicle, because mechanical thresholds of 0.9 % saline and the NTG solvent (6 % propylene glycol, 6 % alcohol, and 0.9 % saline) are the same [26].

### Drug administration

To explore the role of OT and OTR in CM, OT and OTR antagonists were administered to the mice. A solid powder of OT (Med Chem Express/ MCE, USA), an OTR agonist, was dissolved in 0.9 % saline to make a 0.1 mg/ml working solution. For intranasal (i.n) OT administration, the mouse was briefly placed in a supine position with the operator’s left hand. The horizontal position of the head was maintained throughout the procedure to prevent drainage of the drug solution to the trachea and esophagus according to a previous study [27]. Mice were assigned to receive OT (20 µg/kg). Repeated OT treatments were quickly administered at 2 µL per nostril or the same volume of saline every two minutes using the tip of the pipette 30 min before NTG and then once daily at the same time point for 11 days. The selective OTR antagonist (L-368,899 hydrochloride, Sigma, USA), which preferentially binds OTR, was selected. As systemically administered L-368,899 is known to reach the brain, it was intraperitoneally (i.p) injected at a dose of 5 mg/kg once daily 1 h before OT treatment for 11 days [28]. L368,899 was also dissolved in 0.9 % saline according to the manufacturer’s instructions. The drug dosage and methods were conducted according to previous studies [15, 17].

### Behavioral assays to evaluate pain

As headache progresses, the cutaneous allodynia without stimulation occurs in some CM patients [29]. The baseline pain thresholds of periorbit and hindpaw were tested to assess the condition of cutaneous hyperalgesia. All behavioral tests were performed between 09:00 and 15:00 under peaceful conditions. The operator was blinded to the treatment groups and did not participate in the behavioral data analysis. Before the test, the mouse was acclimated to the environment for at least 30 min. Von Frey monofilaments (range from 0.01 to 2 g) were applied perpendicularly to the hind paw or periorbit with the up-down method to assess the mechanical withdrawal threshold as previously described [30, 31].

To assess periorbital mechanical sensitivity, the mouse was placed in a 4 oz. paper cup, with only the head out of the cup. The periorbital region included the caudal regions of the eyes to approximately the midline. Vocalization, quick retraction of the head from stimulation or scratching of the face with the ipsilateral forepaw were considered valid responses.

To assess the mechanical withdrawal threshold of the hind paw, the mouse was separately placed in a suspended acrylic chamber covered with a wire mesh floor. The central area of the plantar surface of the hind paw was selected for testing. Positive responses were defined as lifting or shaking of the paw upon stimulation. The exact force that induced a positive response was recorded. If no positive response occurred, the next higher or lower Von Frey was applied.

For the thermal nociceptive response, the paw withdrawal latency in response to noxious heat stimuli was measured. The mouse was placed in a transparent Plexiglas box. A plantar test apparatus (Techman PL-200, Chengdu, China) recorded the latency time automatically once the mouse lifted its hind paw. A maximum cutoff time of 20 s was set to avoid tissue injury.

All behavioral tests were repeated to achieve at least 3 positive responses for each mouse to obtain the average threshold. Each group contained 6 mice.

### Western Blot (WB) analysis

After the last behavior test on day 11, the mice were deeply anesthetized with sodium pentobarbital (50 mg/kg, i.p.), and the brain tissue of the end of Medulla oblongata end and upper cervical spinal cord (C1 and C2), including the TNC, was harvested immediately and stored at -80 °C. The samples were homogenized in RIPA lysis buffer, containing the protease inhibitor PMSF (Beyotime, Shanghai, China) and phosphatase inhibitors (MCE, USA) for 1 h. The bicinchoninic acid (BCA) protein analysis kit (Beyotime, Shanghai, China) was used to examine the protein concentration. Protein was denatured for 5 min in 100 °C water and stored at − 80 °C. Equal amounts of protein samples (30 µg per lane) were separated on 8 or 10 % SDS polyacrylamide gels (Beyotime, Shanghai, China). After electrophoresis, the proteins were transferred to polyvinylidene difluoride (PVDF) membranes (Millipore, USA). The membranes were blocked for 2 h at room temperature in Tris-buffered saline with Tween-20 (TBST) containing 5 % nonfat milk and incubated overnight at 4 °C with the following primary antibodies: anti-OTR (1:500; Abcam, Cambridge, UK), anti-calcitonin gene-related peptide (CGRP; 1:3000; Abcam, Cambridge, UK), anti-c-fos (1:1000; Abcam, Cambridge, UK), anti-PSD-95 (1:1000; Cell Signaling Technology, Massachusetts, USA), anti-syt-1 (1:1000; Bioss, Beijing, China), anti-snap25 (1:4000; Abcam, Cambridge, UK), anti-NR2B (1:1000; Proteintech, Illinois, USA), anti-NR2B phosphor (pNR2B-Y1472; 1:500; Bioss, Beijing, China), anti-NR2B phosphor (pNR2B-Y1252; 1:1000; Abcam Cambridge, UK), anti-AC1 (1:500; Abcam, Cambridge, UK), anti-PKA C-α (1:3000; Cell Signaling Technology, Massachusetts, USA), anti-CREB (1:1000; Wanlei Bio, Shenyang, China), anti-CREB phosphor (pCREB ; 1:5000; Abcam, Cambridge, UK), and anti-Glyceraldehyde-3-phosphate dehydrogenase (GAPDH; 1:5000; Zen-Bioscience, Chengdu, China). The next day, the membranes were incubated with horseradish peroxidase-conjugated secondary antibodies (goat anti-rabbit, 1:9000; goat anti-mouse, 1:5000; Zen-Bioscience, Chengdu, China) for 1 h at room temperature. The WB bands were visualized with an imaging system (Fusion, Germany) and the ECL Plus Chemiluminescence kit (Zen-Bioscience, Chengdu, China), and quantified with Image J 4.0. The housekeeping protein GAPDH was used to normalize the relative expression level of the target protein. Each group contained 6 mice.

### Immunofluorescence staining

After being deeply anesthetized with sodium pentobarbital (50 mg/kg, i.p.), the mouse was perfused transcardially with 60 ml of cold phosphate-buffered saline (PBS, pH 7.4), followed by 60 ml of 4 % cold paraformaldehyde (PFA) in 0.01 M PBS (pH 7.4). Whole brains, including the TNC, were collected and postfixed overnight with 4 % PFA/0.01 M PBS at 4 °C. Then, 20 and 30 % sucrose/PBS solutions were used sequentially until the tissue sank. The tissue was frozen and sectioned on a cryostat (Leica, Japan) at a thickness of 15 μm. The slices were permeabilized with 0.3 % Triton X-100 for 10 min at room temperature and then blocked with goat serum (Boster, Wuhan, China) for 30 min at 37 °C. Next, the sections were incubated overnight at 4 °C with the following primary antibodies: rabbit anti-OTR (1:100; Abcam, Cambridge, UK), mouse anti-CGRP antibody (1:100; Santa Cruz, California, USA), rabbit anti-c-fos (1:5000; Novus, CO, USA), mouse anti-NeuN (1:500; Novus, CO, USA), and mouse anti-PSD-95 (1:100; Cell Signaling Technology, Massachusetts, USA). On the second day, after rinsing three times for 15 min in PBS, the sections were incubated with corresponding fluorophore-labeled secondary antibodies (conjugated to Alexa Fluor 488, 555, or cy3; 1:500) for 60 min at 37 °C. Then, 4,6-diamidino-2-phenylindole (DAPI) was used to counterstain the nuclei at 37 °C for 10 min. Images were captured with a confocal microscope (LSM800, Zeiss, Germany). The Mouse Brain Atlas was used to identify the TNC region under a low-power field [32]. The fluorescence signal intensity of CGRP was quantified using Image J 4.0. The number of OTR-positive cells and c-fos-positive cells in a square area (field of view, FOV, 320 × 320 µm^2^) centered on the superficial layer of the TNC were assessed with a ×200 objective and quantified by Image J 4.0. Each group contained 4 mice and four to six FOVs per section were investigated.

### Quantitative Real-time Polymerase Chain Reaction (qRT-PCR)

After the mouse was euthanized, the TNC tissue was dissected and stored immediately in liquid nitrogen for qRT-PCR experiments. To determine expression levels of genes, total ribonucleic acid (RNA) was extracted from 30 mg of brain tissue using mRNAiso Plus reagent (TaKaRa, Dalian, China) and quantified by a NanoDrop kit (Thermo, USA). The cDNA synthesis was performed using the PrimeScriptTM RT Kit (TaKaRa, Dalian, China). To quantify the expression of OTR, q-PCR was set up in a 10 µl system using a SYBR Premix Ex Taq II kit (Takara, Dalian, China) and conducted on a CFX-96 Real-Time PCR Detection System (Bio-Rad, Hercules, CA, USA). The primer sequences for OTR and GADPH (Sangon Biotech, Shanghai, China) were as follows OTR (forward primer), 5’- GGTCTCATCAGCTTCAAGATCT-3’; OTR (reverse primer), 5’- ATAAGCTTGACACTACTGACCC-3’; GAPDH (forward primer), 5’-ATGACTCTACCCACGGCAAGC-3’; GAPDH (reverse primer), 5’-GGATGCAGGGATGATGTTCT-3’. The running procedure was 30 s at 95 °C, 45 cycles of 5 s at 95 °C, and 30 s at 57 °C, following a melt curve (65 ~ 95 °C with a heating rate of 0.5 °C and continuous fluorescence measurement. Gene expression was analyzed by the standard ΔΔCq method. Each group contained 6 mice.

### Transmission Electron Microscopy (TEM)

The mouse was anesthetized and perfused transcardially with 60 ml of cold PBS (pH 7.4) and then 60 ml of 2.5 % glutaraldehyde containing 4 % PFA. Then, the tissue was dissected and placed in 2.5 % glutaraldehyde fixative at 4 °C for 24 h. After being cut into 1-mm^3^ pieces with a blade, the TNC tissue was treated with 1 % osmium tetroxide and 1.5 % potassium ferrocyanide. Then, dehydrating, embedding, sectioning, and staining were performed at Chongqing Medical University. The specific steps are described in our previous publication [33]. Images were acquired at 50,000× by a JEM-1400 PLUS transmission electron microscope and analyzed by Image-Pro Plus 6.2. The synaptic interface curvature, the width of the synaptic cleft, and thickness of the postsynaptic density (PSD) of the synaptic ultrastructure were determined as indicators related to synaptic plasticity [34]. Each group contained 4 mice, and four to six images per section were investigated.

### Golgi-cox staining

An FD Golgi Rapid Staining Kit (FD Neuro Technologies, Columbia, MD, USA) was used to assess the morphology of neuronal dendritic spines according to the manufacturer’s instructions [35]. Briefly, after anaesthetization with a lethal dose of sodium pentobarbital, the mouse was executed as soon as possible. The washed TNC tissues were immersed in Rapid Golgi-Cox solution (“Solutions A/B”) for 14 days (the solution was changed once after 24 h) at room temperature with dark light. Next, the tissue was transferred to Solution C for 3 days in the dark at room temperature (changed once after the first 24 h) and sectioned on a vibratome (Leica VT 1200 S, Japan) to obtain 150 μm thick TNC sections. The staining process was as follows. First, free-floating slices were stained in the working solution (1 solution D:1 solution E:2 double-distilled water) for 10 min. Second, the slices were rinsed with double-distilled water(2 times, 4 min per time) and dehydrated with graded ethyl alcohol (50 %, 75 %, 95 %, and 100 %; 4 min for each concentration). Third, xylene was used to make the slices transparent (3 times, 4 min each) and the slices were sealed with a resinous mounting medium for observation. Images were collected with a microscope (Axio Imager A2). Finally, an observer, who was blinded to the treatment conditions, analyzed the number of dendritic spines with Image J. Each group contained 4 mice and four to six images per section were investigated.

### Statistical analysis

Data are reported as mean ± SD. GraphPad Prism version 8.0 (GraphPad Software Inc, San Diego, CA) was used for the statistical analysis and graph generation. Before statistical analysis, the Shapiro-Wilk (S-W) normality test and Bartlett test were applied to conform with normality and homogeneity of variance, respectively. Two-tailed Student’s t-test was performed for two-group comparisons, and one-way ANOVA with Dunnett’s post hoc test was used for multiple comparisons. Two-way ANOVA with the Bonferroni post hoc test was performed for behavioral data because of multiple factors, including drug and time. *P* < 0.05 was considered statistically significant.

### Experimental design

#### Experiment 1

**Fig. 1:**
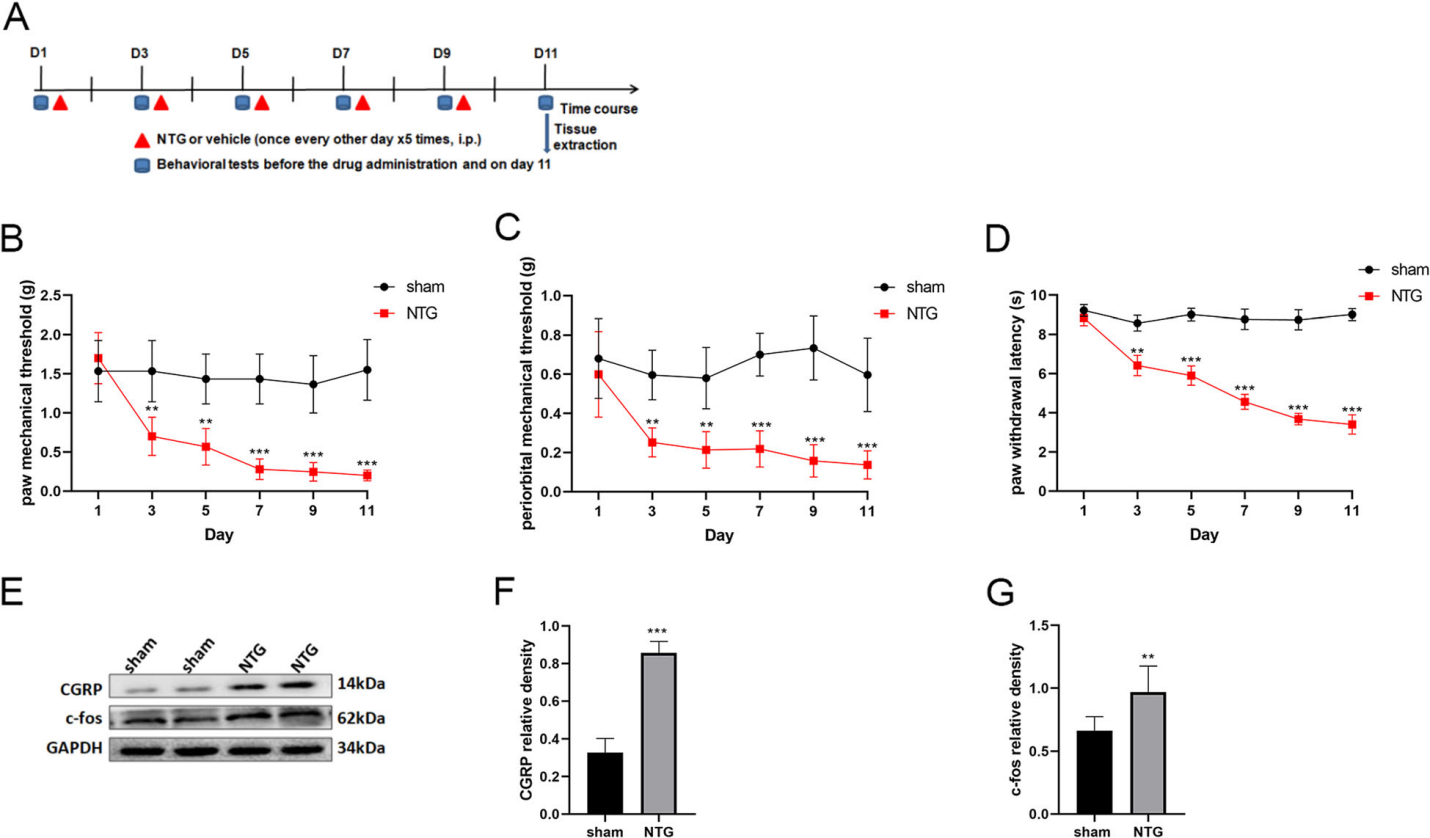
Repeated NTG administration induced basal hyperalgesia and upregulated CGRP and c-fos protein expression. **A** Timeline for the first experiment, D:day. **B**, **C** and **D** The basal mechanical pain threshold of the hind paw (**B**) periorbital area (**C**) and the thermal pain threshold of the hind paw (**D**) were significantly decreased in the NTG group, compared with the sham group beginning on the third day. Two-way ANOVA with the Bonferroni post hoc test, *n* = 6/group, **p < 0.01, ***p < 0.001. **E, F, G** The protein expression of CGRP and c-fos was assessed by WB assay and was markedly higher in the NTG group than in the sham group. Two-tailed Student’s t-test, *n* = 6/group, **p < 0.01, ***p < 0.001

This experiment was performed to assess migraine-associated hyperalgesia after repeated NTG or saline injection. The sham and NTG groups were included. The baseline mechanical withdrawal threshold and thermal nociceptive response in periorbit and hindpaw were tested before each injection of NTG and on day 11. The protein expression of CGRP and c-fos in TNC tissues was also measured to assess the central sensitization. CGRP, which is elevated not only in the TG and TNC of CM animal models but also in the blood and cerebrospinal fluid of CM patients, has been identified as a critical contributing factor for peripheral and central sensitization in the development of CM [36, 37]. Additionally, c-fos, which is a marker of neuron activation, has been recognized as a reliable marker for central sensitization [9]. The expression of OTR at the mRNA and protein levels in TNC was evaluated by qRT-PCR and WB analysis, separately. Immunofluorescence staining was used to observe the morphology and OTR-positive cells. The experimental procedure is shown in Fig. 1A.

#### Experiment 2

This experiment was to assess the effect of OT and the role of OTR in CM mouse model. The sham, NTG + vehicle, NTG + OT, and NTG + OT + L368,899 groups were set up. Briefly, the sham group received saline (i.p.) 1 h before the next drug, saline (i.n.) 30 min before the next drug, and saline (i.p.). The NTG + vehicle group received saline (i.p.) 1 h before the next drug, saline (i.n.) 30 min before NTG, and NTG (i.p.). The NTG + OT group received saline (i.p.) 1 h before OT, OT (i.n.) 30 min before NTG, and NTG (i.p.). The NTG + OT + L368,899 group received L368,899 (i.p.) 1 h before OT, OT (i.n.) 30 min before NTG, and NTG (i.p.). The same volume of the drug was given at each stage. The dosage and methods are described in the Method section. The assessment of pain threshold was consistent with experiment one. TNC tissues were harvested for WB, immunofluorescence, TEM, and Golgi-cox staining to evaluate the related targets and neuronal morphology. The experimental procedure is shown in Fig. 3A.

## Results

### Recurrent NTG injection induced hyperalgesia and upregulation of CGRP, c-fos in TNC

In experiment 1, the baseline mechanical thresholds (hind paw and periorbit) and thermal nociceptive responses declined gradually on days 3, 5, 7, and 9 before drug administration in the NTG group compared with the sham group. On day 11, the pain thresholds in the NTG group remained lower than those in the sham group (Fig. 1B, C, D). In addition, there was no significant difference in pain threshold among the groups on day 1. Compared with that in the sham group, the protein expression of CGRP and c-fos, was significantly increased in the NTG group (Fig. 1E, F, G). These behavioral data and the CGRP and c-fos expression data indicated that central sensitization occurred after repeated NTG injection.

### OTR was upregulated in the TNC after recurrent NTG administration

**Fig. 2:**
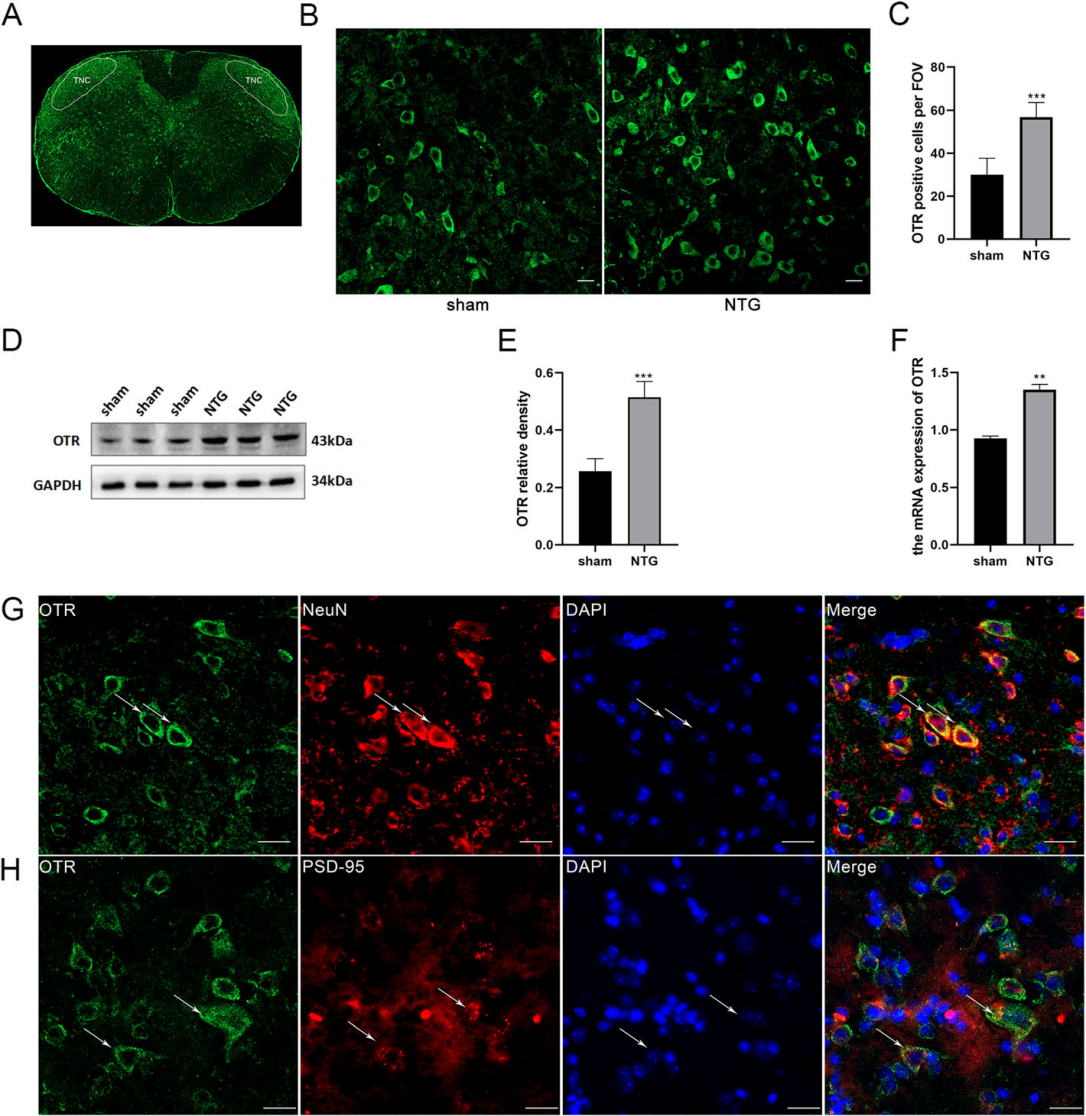
The increase of OTR in CM mouse and the distribution of OTR in the TNC. **A** The white full line frame indicates the TNC area stained for OTR. **B** Representative images of OTR immunoreactivity in the sham and NTG groups. **C** The average number of OTR-positive cells in the NTG group was significantly increased compared to that in the sham group (scale bar: 20 μm). **D** and **E** WB analysis showed that OTR protein expression in the NTG group was significantly increased compared with that in the sham group. **F** The mRNA expression of OTR in the NTG group was statistically higher than that in the sham group. **G** Double immunofluorescence labeling of OTR (green) and NeuN (red). In the TNC, most OTR was distributed in neurons (scale bar: 50 μm). **H** Double immunofluorescence labeling of OTR (green) and PSD-95 (red). Some OTR was colocalized with the postsynaptic membrane in the TNC (scale bar: 50 μm). Two-tailed Student’s t-test, *n* = 4/group for immunofluorescence analysis, *n* = 6/group for WB analysis; **p < 0.01, ***p < 0.001 vs. the sham group

To investigate the effect of NTG treatment on OTR, we measured the expression of OTR in the sham and NTG groups by WB analysis and qRT-PCR. The protein and RNA levels of OTR were significantly increased in the NTG group compared with the sham group (Fig. 2D, E, F). The immunofluorescence staining results showed that the number of OTR-positive cells per FOV in the TNC area of the NTG group was significantly higher than that in the sham group (Fig. 2B, C). In addition, double immunofluorescence staining showed that OTR was abundantly expressed in neurons, and OTR was observed in numerous small and medium-sized neurons, mainly in the cytomembrane with a small amount in the cytoplasm (Fig. 2G). Moreover, another double immunofluorescence staining indicated that OTR was partially colocalized with the PSD-95, a marker of the postsynaptic membrane (Fig. 2H). Based on these results, we concluded that OTR expression was upregulated in the TNC after repeated NTG injection and might be involved in the pathophysiological mechanism of CM.

### OT ameliorated pain sensitization and reduced neuronal activation in CM mouse model

**Fig. 3:**
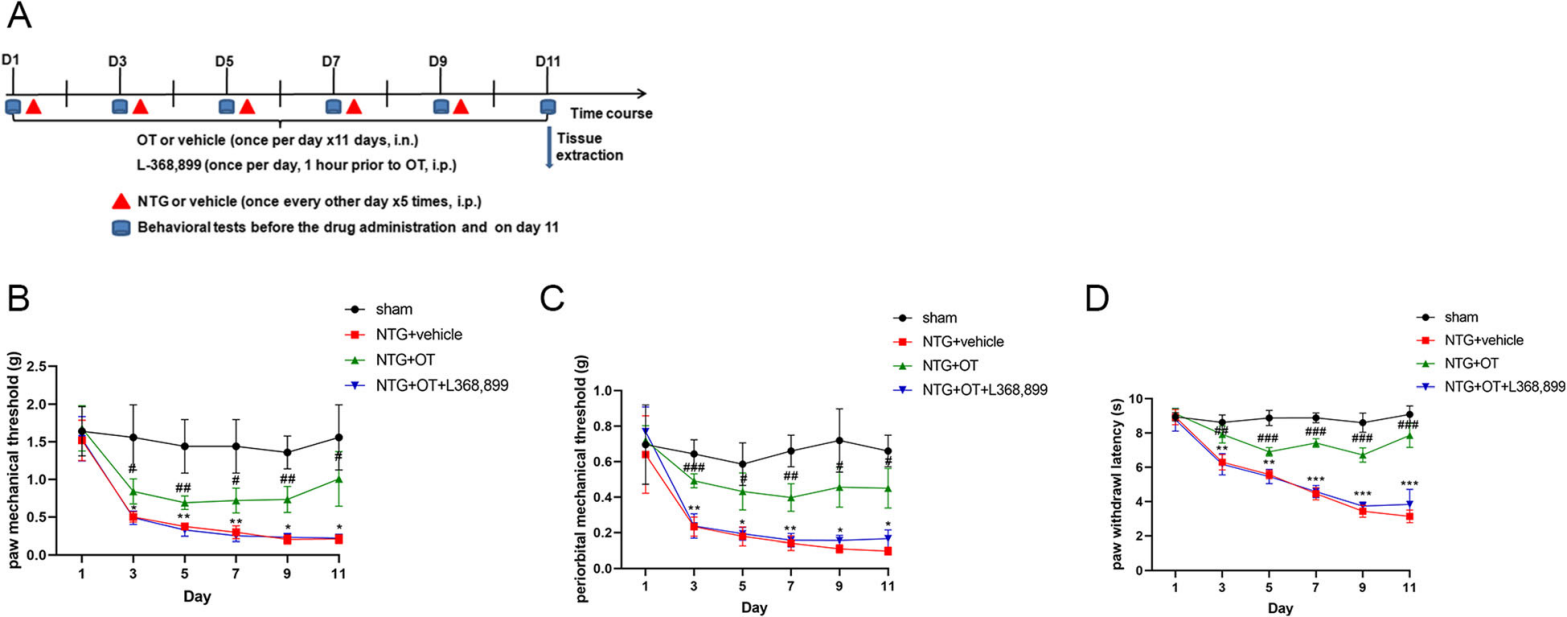
OT ameliorated pain sensitization and reduced neuronal activation via OTR. **A** Timeline for the second experiment. Note that L-368,899 was intraperitoneally injected daily 1 h before OT treatment. The mechanical and thermal pain thresholds of the hind paw and periorbital area were assessed before each NTG injection and on day 11. **B**, **C**, **D** The basal mechanical pain thresholds of the hind paw (**B**), periorbital area (**C**), and the thermal pain response of the hind paw (**D**) were significantly increased in the NTG + OT group compared with the NTG + vehicle group. The pain threshold in the NTG + OT + L368,899 group was markedly decreased compared with the NTG + OT group. Moreover, there was no significant difference between the NTG + vehicle and NTG + OT + L368,899 groups. Two-way ANOVA with the Bonferroni post hoc test, *n* = 6/group; #*p* < 0.05, ##*p* < 0.01, ###*p* < 0.001 vs. the NTG + vehicle; *p < 0.05, **p < 0.01, ***p < 0.001 vs. the sham group

**Fig. 4:**
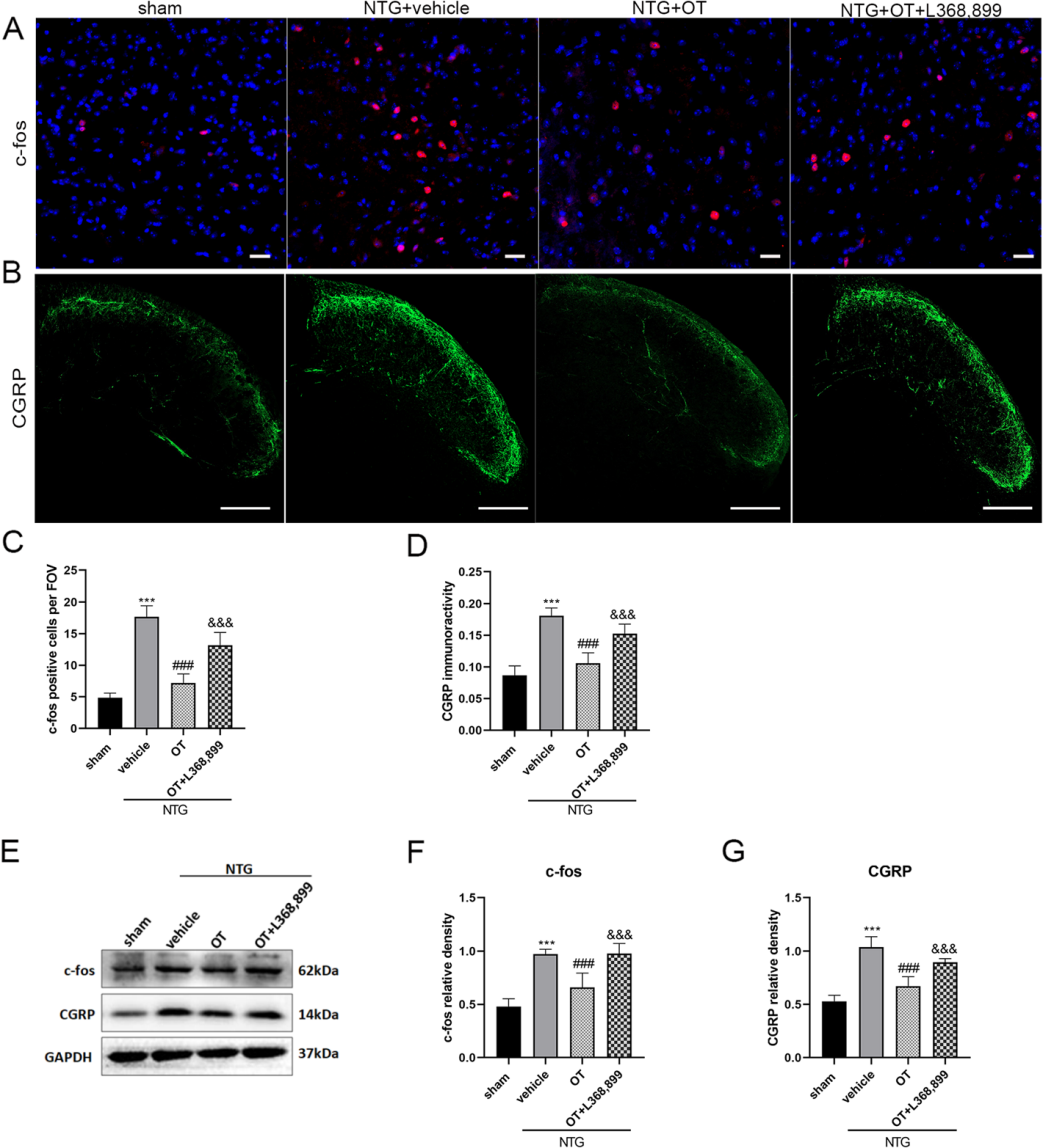
Effect of OT on CGRP and c-fos expression levels. **A** and **C** show c-fos, **B** and **D** show CGRP. Immunofluorescence staining showed that the number of c-fos-positive cells per FOV and the average fluorescence intensity of CGRP were significantly increased in the NTG + vehicle group compared with the sham group. OT administration significantly reduced the expression of CGRP and c-fos, while these effects were prevented after adding L368,899 (scale bars: 20 μm for c-fos, 100 μm for CGRP). **E**, **F** and **G** WB data quantification showed similar results to the immunofluorescence staining results. One-way ANOVA with Dunnett’s post hoc test, *n* = 4/group for immunofluorescence analysis, *n* = 6/group for WB analysis; ***p < 0.001 vs. the sham group; ###*p* < 0.001 vs. the NTG + vehicle group; &&&*p* < 0.001 vs. the NTG + OT group

In experiment 2, behavior assessments showed that the mechanical and thermal pain responses of the hind paw and periorbital area in the NTG + OT group were significantly increased compared with the NTG + vehicle group. The pain thresholds in the NTG + OT + L368,899 group were markedly decreased compared with the NTG + OT group (Fig. 3B, C, D). In addition, the number of c-fos-positive cells in the superficial layers of the TNC (Fig. 4A, C) and the mean optic density (OD) of CGRP immunoreactive fiber were reduced by OT administration (Fig. 4B, D).WB analysis showed that the protein levels of c-fos and CGRP were lower in the NTG + OT group than in the NTG + vehicle group (Fig. 4E, F, G). However, the expression levels of c-fos and CGRP were significantly increased after adding L-368,899 (Fig. 4). These results indicated that OT alleviated the central sensitization of CM mouse model via OTR in the TNC.

### OT treatment decreased the overexpression of phosphorylated NR2B in CM mouse model

**Fig. 5:**
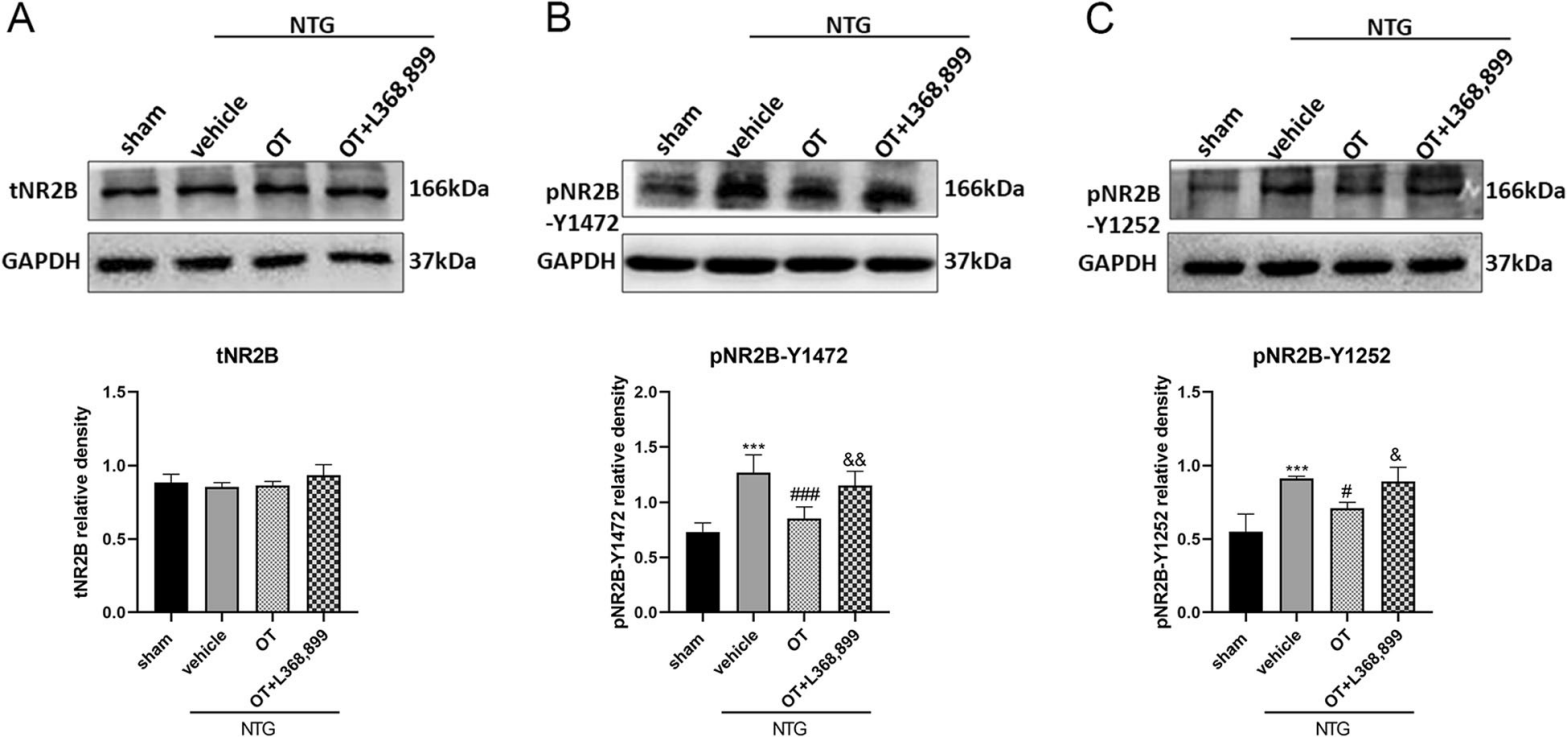
Effect of OT on phosphorylated NR2B expression levels. **A** Representative WB bands showed that there was no significant difference in total NR2B expression among the four groups. **B** and **C** The protein levels of pNR2B-Y1472 (**B**) and pNR2B-Y1252 (**C**) were significantly elevated in the NTG + vehicle group compared with the sham group. After OT treatment, the expression levels of these two proteins were significantly reduced. These effects of OT were inhibited by L368,899. One-way ANOVA with Dunnett’s post hoc test, *n* = 6/group; ***p < 0.001 vs. the sham group; #*p* < 0.05, ###*p* < 0.001 vs. the NTG + vehicle group; &*p* < 0.05, &&*p* < 0.01 vs. the NTG + OT group

Phosphorylated NR2B may affect the recruitment of NMDA receptors, and control the number and function in the cell membrane [21]. To determine whether OT influences NMDA receptor function, the protein expressions of total NR2B, pNR2B-Y1472, and pNR2B-Y1252 were measured by WB analysis. Our results showed that the expression of pNR2B-Y1472 and pNR2B-Y1252 in the NTG + vehicle group was significantly increased compared to the sham group. However, repeated OT administration significantly down-regulated the phosphorylation of NR2B, and L368,899 prevented the effect of OT (Fig. 5B, C). In addition, the expression of total NR2B was not different among the four groups (Fig. 5A).

### OT treatment reduced the overexpression of synapse-associated proteins PSD95, syt-1, and snap25 in CM mouse model

**Fig. 6:**
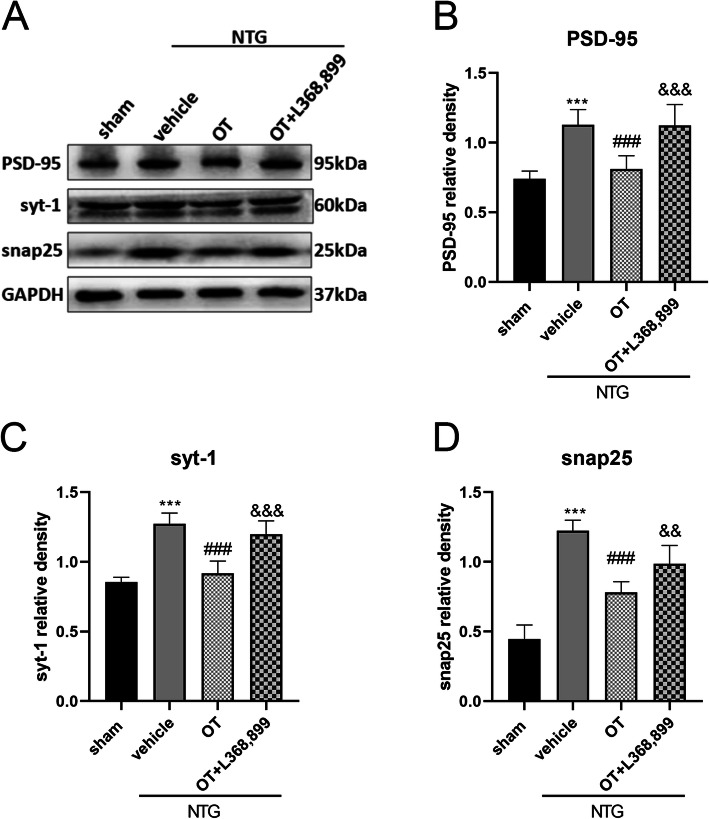
Effect of OT on synapse-associated protein expression levels. **A** Representative WB bands showing the expression of PSD-95, syt-1, and snap25. **B**, **C**, and **D** Quantification of PSD-95 (**B**), syt-1 (**C**), and snap25 (**D**) protein levels showed that these proteins were significantly increased in the NTG + vehicle group compared to the sham group. The expression levels of these three proteins after OT treatment were reduced, while L368,899 blocked these effects. One-way ANOVA with Dunnett’s post hoc test, *n* = 6/group; ***p < 0.001 vs. the sham group; ###*p* < 0.001 vs. the NTG + vehicle group; &&*p* < 0.01, &&&*p* < 0.001 vs. the NTG + OT group

As phosphorylated NR2B participates in the regulation of synaptic plasticity, which is vital in the development of CM [23], we further explored whether OT could regulate the expression of PSD-95, syt-1, and snap25, which are synapse-associated proteins. PSD-95 is essential in activity-driven synapse stabilization and interacts with G protein-coupled receptors [38, 39]. Syt-1 is considered to be the major Ca^2+^ sensor in cell secretion and participates in the discharge and transport of synaptic vesicles [40]. Snap25 is involved in the exocytosis of postsynaptic glutamate receptors under basal conditions and long-term synaptic plasticity [41]. WB analysis revealed that the expression of PSD-95, syt-1, and snap25 in the NTG + vehicle group was significantly increased, compared with that in the sham group, while repeated OT administration significantly decreased the expression of these three proteins in the NTG + OT group. Moreover, the effect of OT was inhibited in the NTG + OT + L368,899 group (Fig. 6).

### OT treatment restored the aberrant synaptic plasticity in CM mouse model

**Fig. 7:**
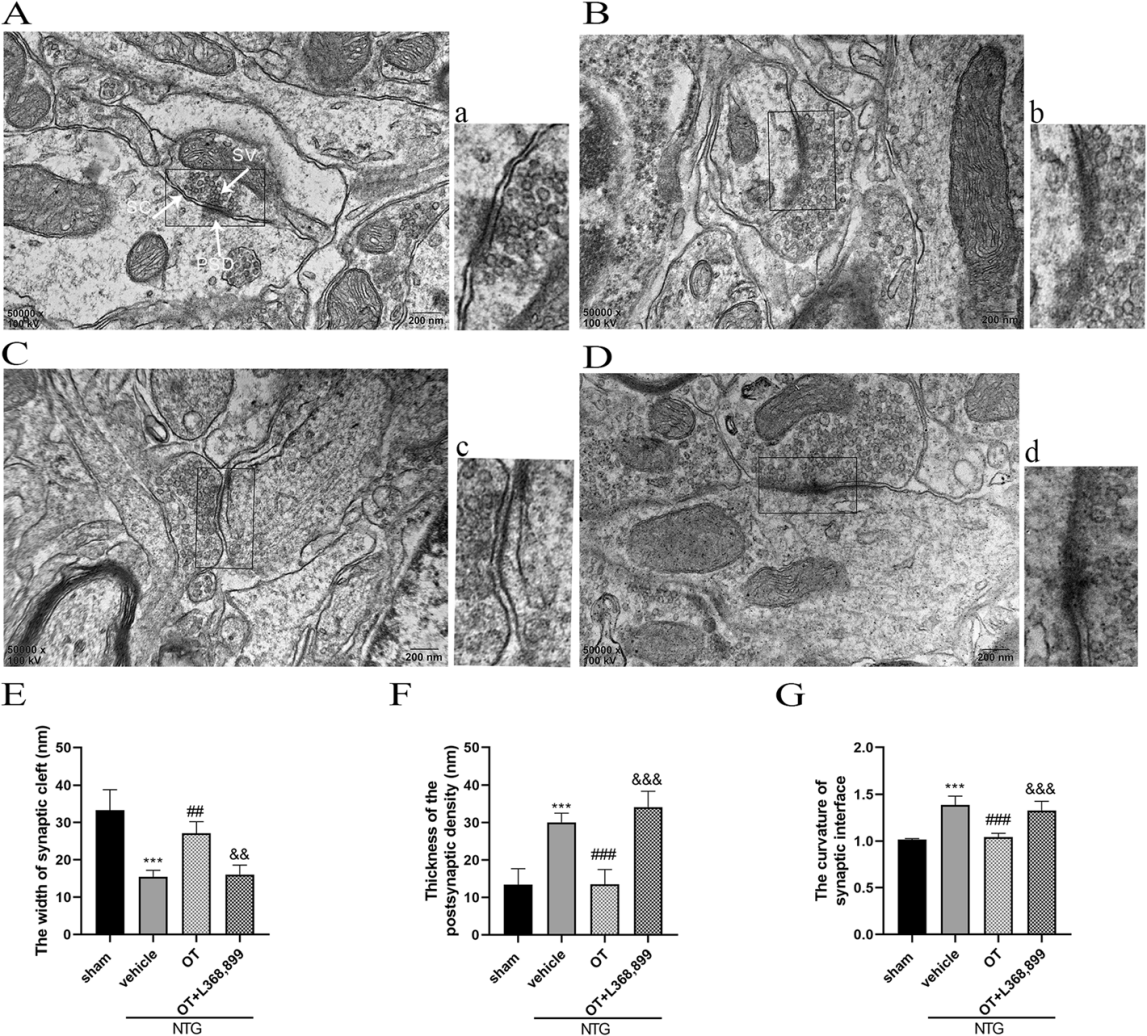
Representative images and quantitative analysis of the synaptic ultrastructure in the TNC. **A** and **a** show the sham group, **B** and **b** show the NTG + vehicle group, **C** and **c** show the NTG + OT group, **D** and **d** show the NTG + OT + L368,899 group. **a**-**d** Magnification of the rectangle frame from each group was used to quantify parameters of the synaptic interface in the four groups. The width of the synaptic cleft (**E**) was decreased, and the thickness of PSD (**F**) and the synaptic interface curvature (**G**) were significantly increased in the NTG + vehicle group compared to the sham group. In the NTG + OT group, these abnormal alterations were alleviated. The morphological parameters in the NTG + OT + L368,899 group were similar to those in the NTG + vehicle group. One-way ANOVA with Dunnett’s post hoc test, *n* = 4/group; **p < 0.01, ***p < 0.001 vs. the sham group; ##*p* < 0.01, ###*p* < 0.001 vs. the NTG + vehicle group; &&*p* < 0.01, &&&*p* < 0.001 vs. the NTG + OT group; scale bars: 200 nm; PSD, postsynaptic density; SC, synaptic cleft; SV, synaptic vesicle

The structural and functional alterations of synapses are closely related to synaptic plasticity. In Fig. 7, we showed the representative images of the synaptic structure in the TNC as observed by TEM. Among these figures, the synaptic cleft in the sham and NTG + OT groups was visible, and the PSD was appropriate. However, the synaptic cleft and PSD in the NTG + vehicle and NTG + OT + L368,899 groups were constricted and blurred compared with those in the sham group. In addition, the curvature of the synaptic interface was augmented in the NTG + vehicle and NTG + OT + L368,899 groups to enhance the synaptic transmission efficiency (Fig. 7A-D and a-d).

Statistical analysis showed that the width of the synaptic cleft (Fig. 7E) was decreased in the NTG + vehicle group, compared with the sham group. The PSD thickness (Fig. 7F) and synaptic interface curvature (Fig. 7G) in NTG + vehicle group were significantly increased, compared with the sham group. After repeated OT treatment, these abnormal alternations were alleviated. Moreover, L368,899 blocked the effect of OT.

**Fig. 8:**
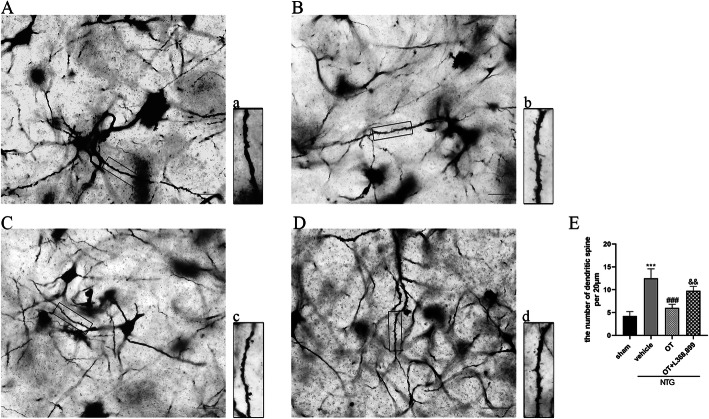
Representative images and quantitative analysis of the dendritic spines in the TNC. **A** and **a** show the sham group, **B** and **b** show the NTG + vehicle group, **C** and **c** show the NTG + OT group, **D** and **d** show the NTG + OT + L368,899 group. **a-d** Magnification of the rectangle frame from each group was used to quantify the spine density in the four groups. **E** Golgi-Cox staining showed that the number of dendritic spines per 20 μm was significantly higher in the NTG + vehicle group than in the sham group. The number of dendritic spines after OT treatment was reduced, while the effect of OT was inhibited by L368,899. One-way ANOVA with Dunnett’s post hoc test, *n* = 4/group; ***p < 0.001 vs. the sham group; ###*p* < 0.001 vs. the NTG + vehicle group; &&*p* < 0.01 vs. the NTG + OT group; scale bars: 20 μm

In addition, the Golgi staining results also showed that the number of dendritic branches in the NTG + vehicle group was greater than that in the sham group. After OT treatment, the number of the dendritic branch was significantly decreased. The OT-mediated effect was prevented when L368,899 was added (Fig. 8). These results suggested that repeated OT treatment might inhibit the enhanced synaptic plasticity of spinal dorsal horn neurons induced by NTG in CM mouse.

### The AC1/PKA/pCREB pathway was depressed after OT treatment

**Fig. 9:**
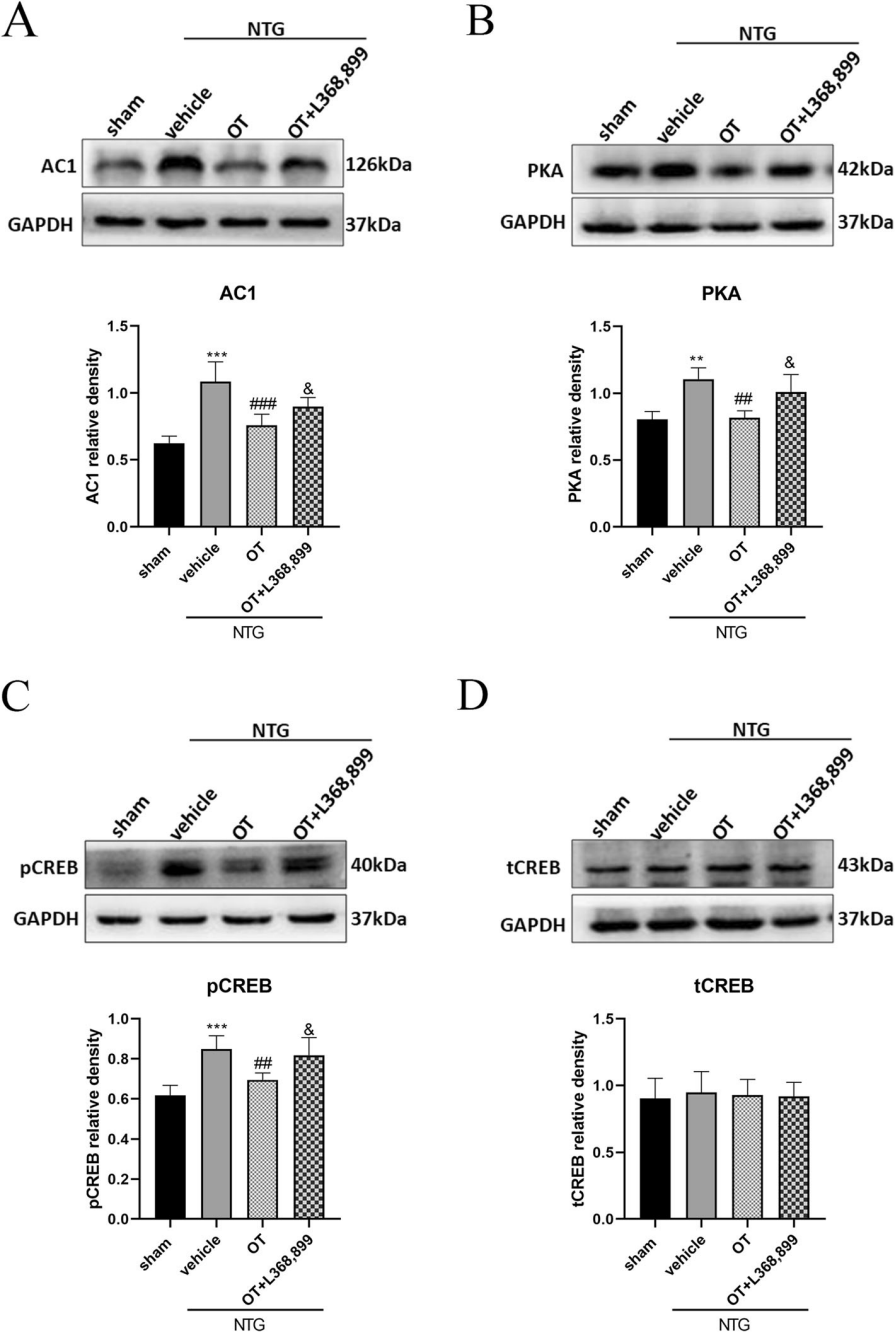
Effect of OT on the AC1/PKA/pCREB pathway. **A**, **B** and **C** WB results showed that the protein levels of AC1 (**A**), PKA (**B**), and pCREB (**C**) were significantly elevated in the NTG + vehicle group compared with the sham group. The expression levels of these three proteins after OT treatment were reduced, while L368,899 prevented the effect of OT. **D** There was no significant difference in CREB expression among the four groups. One-way ANOVA with Dunnett’s post hoc test, *n* = 6/group; **p < 0.01, ***p < 0.001 vs. the sham group; ##*p* < 0.01, ###*p* < 0.001 vs. the NTG + vehicle group; &*p* < 0.05 vs. the NTG + OT group

In vivo and in vitro studies show that the AC1/PKA/pCREB pathway plays an important role in reinforcing the NMDA receptor functions by positive cellular feedback in chronic pain [21, 42]. We investigated whether the AC1/PKA/pCREB signaling was a potential pathway involved in OT/OTR-mediated regulation of NMDA receptor function. In the NTG + vehicle group, after NTG injection, the protein expression levels of AC1, PKA, and pCREB were significantly increased compared to that in the sham group, while repeated treatment with OT reduced the protein levels of AC1, PKA, and pCREB. However, L368,899 restrained the effect of OT (Fig. 9A, B, C). In addition, the protein expression level of total CREB was not altered in the four groups (Fig. 9D). These results demonstrated that the AC1/PKA/pCREB pathway might be associated with the OT-mediated decline of NMDA receptor function.

## Discussion

In the present study, we explored the preventive effect of OT in CM mouse model and elucidated the potential mechanisms involved. We observed that the OTR expression in the TNC of CM mouse was significantly increased, and the repeated intranasal OT administration could elevate the decreased baseline thresholds induced by NTG and alleviate central sensitization in the TNC. Furthermore, OT could regulate the synaptic plasticity by inhibiting the overexpression of phosphorylated NR2B, synaptic plasticity-related proteins, and restoring the abnormal synaptic structural changes. The AC1/PKA/pCREB pathway, which was downstream of OTR and regulated the NMDA receptor function, was also suppressed after OT treatment, suggesting that this pathway is a possible mechanism underlying the OT-mediated effect. However, these effects of OT were prevented by an OTR antagonist. These results were consistent with our hypothesis that OT could alleviate central sensitization by regulating synaptic plasticity via OTR.

In the first part of our study, we established a reliable CM model by recurrent NTG intraperitoneal injection, that mimicked the progression of migraine. The observation of gradually decreased mechanical and thermal thresholds before NTG injection, especially on day 11 without NTG injection, implied hypersensitivity in the cranial and extracephalic regions, which is similar to the previous studies [25, 43]. The overexpression of CGRP and c-fos in TNC after repeated NTG injection also implies that the reliable central sensitization can be constructed successfully.

To elicit its actions in the brain, OT must activate its main target, OTR. Evidence suggests that the different exogenous OT delivery methods may affect analgesia efficiency [44]. As OT in the blood cannot pass through the blood-brain barrier, intranasal OT administration, which is currently the preferred method in clinical trials targeting the central nervous system (CNS), may increase OT concentrations in CNS via channels surrounding trigeminal and olfactory nerve fibers [15, 45]. The quantification of OT in brain regions by immunoassays and in cerebrospinal fluid by ELISA techniques after intranasal OT administration have shown that intranasal administration OT can enter the brain, especially the regions in the trigeminal nervous system, such as the TG and TNC [15, 24]. OT is also found to increase, with peak levels occurring 30 ~ 60 min after nasal administration in both the hippocampus and amygdale by microdialysates analysis [27]. So, we selected intranasal OT delivery to conduct our experiments. Our study showed that the repeated administration of OT significantly alleviated the mechanical and thermal hyperalgesia induced by recurrent NTG injection. In addition, OT also remarkably reduced the expression of CGRP and c-fos in the TNC, indicating the alleviation of central sensitization. Moreover, the effect of OT was inhibited when a specific OTR antagonist was pretreated. Although OTR and vasopressin (V1A) receptors have similar affinities to OT, previous studies support the role of OTR in inhibiting the nociceptive response at the spinal level [46, 47]. Therefore, the above results illustrated that repeated intranasal OT administration may be a reliable way to alleviate central sensitization via OTR in the TNC.

In addition, the upregulation of OTR is found under pathological states. Previous studies have shown that OTR is overexpressed in the TG after complete Freund’s adjuvant administration into the temporomandibular joint or prolonged electrocutaneous stimulation of the cheek [46]. A similar increase of OTR in the TG and TNC was observed in a model of inferior alveolar nerve injury [48]. We demonstrated the upregulation of OTR in the TNC after NTG injection and confirmed the relationship between OTR and CM. These endogenous increases of OTR may also reflect an endogenous protective effect after nociceptive stimulus [49]. However, the precise mechanism is not clear.

Under different pathological conditions such as chronic pain and stroke, postsynaptic NMDA receptors induce plasticity changes, including long-term potentiation (LTP) and long-term depression (LTD) [22, 50]. Central sensitization occurred in CM is characterized by increased excitability and synaptic strength [23]. NMDA receptor activation seems to be crucial for the induction of central sensitization [20]. Phosphorylation of tyrosine on the NR2B subunit reduces the endocytosis of NR2B-containing NMDARs to further increase the influx of calcium and enhance NMDA receptor functions [22]. Our double immunofluorescence staining results showed that OTR colocalized with PSD, indicating that OTR could exert a postsynaptic effect. Our WB results showed that OT inhibited the phosphorylated NR2B in the TNC. The postsynaptic inhibitory effect of OT between the primary afferent fibers and the dorsal horn neuron is also found in Robinson’s study [51]. An electrophysiological study also demonstrates that OT-mediated inhibition of TRPV1 activation in the superficial dorsal horn of the spinal cord is postsynaptic [52]. Consistent with our results, a study shows that OT could prevent the activation of neurons induced by NMDA on spinal cord dorsal horn slices in vitro [53]. These findings illustrated that repeated OT could inhibit the expression of phosphorylated NR2B in postsynapse to reduce the NMDA receptor functions.

This aberrant synaptic plasticity is found in neuropathic pain and central poststroke pain [54, 55]. In our previous study, the synaptic ultrastructure and the number of dendritic branches in the TNC of CM animal models are significantly changed [23]. As mentioned above, OTR was partially expressed in the postsynaptic membrane of neurons and it also appeared to be a relevant factor for the regulation of synapse-associated proteins. Our results showed that OT treatment could decrease the expression of synaptic plasticity-related proteins and ameliorate the abnormal synaptic ultrastructure and dendritic branches induced by NTG. However, a study has shown that OTR knockout mice suffer from a reduction in PSD-95 [56]. This is because the functionality of the OTR system can promote the formation and stability of the synapse in the development of the brain, which is different from the pathological state of pain [57]. In short, the aberrant synaptic plasticity in CM could be restored to normal after repeated OT administration.

As OTR belongs to the G protein-coupled receptor superfamily, the long-term OT treatment could activate Gαi proteins of OTR and inhibit AC1 activity [58]. However, the activation of the AC1/PKA/pCREB pathway in acute and chronic pain induces postsynaptic LTP to enhance synaptic transmission efficiency, thus resulting in central sensitization [59, 60]. Our results showed that the protein expression levels of AC1, PKA, and pCREB were increased in the NTG group compared with the sham group, while OT treatment reduced the expression of these proteins. This finding indicated that the AC1/PKA/pCREB signaling pathway might be depressed after the activation of OTR by OT treatment. While previous studies have reported that OT may activate gamma-aminobutyric acid (GABA)ergic interneurons in the spinal dorsal horn and promote GABA release to inhibit the function of postsynaptic glutamate receptors, including NMDA receptors [52, 61]. A recent study has demonstrated that long-term treatment with OT can downregulate the expression of PKA and pCREB in neonatal cardiomyocytes [58]. Comparison of the different mechanisms mentioned above implies that the effect of long-term treatment with OT may be different from the short-term or single-dose reatment. Our observation of OT treatment-induced downregulation of AC1, PKA, and pCREB may be the long-term effect. However, whether GABAergic interneurons are involved in the long-term effect of OT treatment needs to be explored further. In addition, the direct and indirect effect of OT remain to be illustrated by further electrophysiological experiments.

## Conclusions

In summary, our study demonstrates that repeated intranasal OT administration can prevent hyperalgesia behavior and central sensitization by regulating synaptic plasticity via OTR in CM mouse model. Furthermore, the AC1/PKA/pCREB signaling pathway may be involved in the protective effect of OT. Therefore, repeated intranasal OT may be a potential candidate for CM prevention.

## Data Availability

The data used in this article are available if necessary.

## Abbreviations

CM: Chronic migraine
EM: Episodic migraine
OT: Oxytocin
OTR: Oxytocin receptor
TG: Trigeminal ganglion
TNC: Trigeminal nucleus caudalis
NR2B: N-methyl D-aspartate receptor subtype 2B subunit
NMDA: N-methyl-D-aspartate
AC1: Adenylyl cyclase 1
PKA: Protein kinase A
CREB: Cyclic adenosine monophosphate response element-binding protein
NTG: Nitroglycerin
CGRP: Calcitonin gene-related peptide
PSD-95: Postsynaptic density protein 95
syt-1: Synaptophysin-1
snap25: Synaptosomal-associated protein
GAPDH: Glyceraldehyde-3-phosphate dehydrogenase
qRT-PCR: Quantitative real-time polymerase chain reaction
WB: Western blot
i.n: Intranasal
i.p: Intraperitoneal
PBS: Phosphate-buffered saline
PFA: Paraformaldehyde
FOV: Field of view
TEM: Transmission electron microscope
GABA: Gamma-aminobutyric acid
